# Osteocytic FGF23 and Its Kidney Function

**DOI:** 10.3389/fendo.2020.00592

**Published:** 2020-08-28

**Authors:** Rafiou Agoro, Pu Ni, Megan L. Noonan, Kenneth E. White

**Affiliations:** ^1^Department of Medical and Molecular Genetics, Indiana University School of Medicine, Indianapolis, IN, United States; ^2^Medicine/Division of Nephrology, Indiana University School of Medicine, Indianapolis, IN, United States

**Keywords:** osteocyte, FGF23, FGF23 signaling, Klotho, kidney

## Abstract

Osteocytes, which represent up to 95% of adult skeletal cells, are deeply embedded in bone. These cells exhibit important interactive abilities with other bone cells such as osteoblasts and osteoclasts to control skeletal formation and resorption. Beyond this local role, osteocytes can also influence the function of distant organs due to the presence of their sophisticated lacunocanalicular system, which connects osteocyte dendrites directly to the vasculature. Through these networks, osteocytes sense changes in circulating metabolites and respond by producing endocrine factors to control homeostasis. One critical function of osteocytes is to respond to increased blood phosphate and 1,25(OH)_2_ vitamin D (1,25D) by producing fibroblast growth factor-23 (FGF23). FGF23 acts on the kidneys through partner fibroblast growth factor receptors (FGFRs) and the co-receptor Klotho to promote phosphaturia *via* a downregulation of phosphate transporters, as well as the control of vitamin D metabolizing enzymes to reduce blood 1,25D. In the first part of this review, we will explore the signals involved in the positive and negative regulation of FGF23 in osteocytes. In the second portion, we will bridge bone responses with the review of current knowledge on FGF23 endocrine functions in the kidneys.

## Introduction

The mammalian skeleton is formed by several types of bone which interconnect to provide structural support for the body. In adult humans, the reference value for the skeleton weight is 10.5 kg (1, 2) representing up to 15% of the average body weight. Bone mass is dynamically regulated during a lifetime, and subjected to changes with uncontrollable parameters such as age, gender, genetics, and ethnicity; as well as controllable factors such as lifestyle behaviors including physical activity levels, smoking and alcohol consumption patterns, and diet (3, 4). Beyond its important role to enable mobility and provide needed support and structure to the body, bone represents an important reservoir of several minerals including phosphate and calcium, both of which are required for proper mineral metabolism and cellular functions (5).

Bone is a mineralized connective tissue formed with three primary cell types that direct intrinsic skeletal properties: osteoclasts, which resorb mineralized bone; osteoblasts, which form the bone matrix; and osteocytes, which are considered terminally differentiated osteoblasts (6, 7). Although morphologically and functionally distinct in the bone, osteoclasts, osteoblasts, and osteocytes are interdependent and produce growth factors to support each other's functions as well as responding in a coordinated manner to metabolic demands, physical stimuli, and structural duties (8). The three main bone cells are derived from two distinct lineages; osteoblasts and osteocytes derive from pluripotent mesenchymal stem cells and share the same progenitors as fibroblasts and adipocytes (9, 10), whereas osteoclasts are derived from hematopoietic progenitors in the monocyte and macrophage lineage (11–13).

The osteocytes, which represent the majority of adult skeletal cells, are deeply embedded with abilities to communicate locally with osteoblasts and osteoclasts. This function is necessary to control skeletal formation as orchestrated by osteoblasts, and bone resorption dictated by osteoclasts, as well as controlling the physiological function of distant organs such as the kidney. Taking advantage of its dendrites, which connect to the vasculature and give these atypical cells direct access to the circulation, the osteocyte can send and receive signals with the vascularized organs. Among the important osteocyte-secreted factors is fibroblast growth factor-23 (FGF23). Once produced and secreted by osteocytes, this hormone preferentially acts on kidney and parathyroid glands to regulate phosphate and vitamin D homeostasis. FGF23 activity mainly occurs through the binding of FGF23 to FGF receptor (likely FGFR1), which requires the presence of its membrane and/or soluble co-receptor Klotho for a potent FGF23-induced downstream signaling cascade (14). In this review, the stimulative and repressive regulatory mechanisms involved in FGF23 production and processing in osteocytes will be discussed. We will also bridge the control of FGF23 in osteocytes with highlighting the key signaling pathways involved in phosphate, 1,25D, calcium, and sodium metabolism induced by FGF23 in the kidneys.

## The Osteocyte: A Critical Bone and Endocrine Cell

As the most prevalent cell in bone, osteocytes have important roles both within, and outside the skeleton. The osteocytes are considered as major orchestrators of skeletal activity; these cells can sense and integrate mechanical and chemical stimuli from the microenvironment with the goal to properly regulate bone formation and resorption. Osteocytes derive from mature and matrix-producing terminally differentiated osteoblasts (6). During their last phase of differentiation, mature osteoblasts become embedded in the matrix and generate cellular extensions, which are future osteocyte dendrites. To establish a sophisticated and complex network called the lacunocanalicular system, the dendrites of the newly formed osteocytes are fastened with the dendrites of existing osteocytes through a multitude of canaliculi (15). Even after the terminal differentiation of mature osteoblasts to generate osteocytes, the latter remain active in contributing to bone remodeling. For instance, osteocytes produce sclerostin (SOST), which binds to low-density lipoprotein receptor-related protein (Lrp)5/6, and neutralizes the anabolic Wnt/beta-catenin pathway (16, 17), thus negatively regulating bone formation. Osteocytes also produce the receptor activator of nuclear factor-κB ligand (RANKL) which stimulates osteoclastogenesis, thus promoting bone resorption (18, 19).

In bone, osteocytes are bathed in canalicular fluid that delivers and exchanges nutrients, circulating factors, mechanical signals, and oxygen between the circulation and the “fixed and embedded” osteocytes (20). During the last decade, the osteocyte lacunocanalicular network has gained tremendous attention because of accumulating and convincing evidence describing osteocytes as a major endocrine cell, and its role in the production of critical hormones targeting several organs. One of the most important osteocyte-secreted factors is FGF23. This hormone was first characterized as a mammalian “phosphatonin” by identifying stabilizing mutations in the *FGF23* gene in patients with autosomal dominant hypophosphatemic rickets (ADHR), a renal phosphate wasting disorder (21).

## FGF23 Production and Cleavage in Osteocytes

FGF23 is a phosphaturic hormone derived and secreted primarily by bone osteocytes. Mature, bioactive FGF23 is physiologically designed to target the kidney to regulate phosphate and vitamin D homeostasis; and, in a feedback mechanism to control bone mineralization and FGF23 production (22, 23). The mechanisms of FGF23 regulation in osteocytes are not fully understood. Several breakthroughs have been made that greatly improved our knowledge on the mechanisms of osteocytic FGF23 upregulation or downregulation through differential signaling pathways, as well as the pathophysiological response to multiple stimuli. In addition to several mechanisms involved in the transcriptional regulation of FGF23, another layer of FGF23 regulation in bone is the ability of the mature protein to be proteolytically cleaved within osteocytes to generate inactive FGF23 fragments before its secretion into the bloodstream (24).

### Regulation of Osteocytic FGF23 by Phosphate, FGF23, FGFR Activation, and Klotho

**Figure 1:**
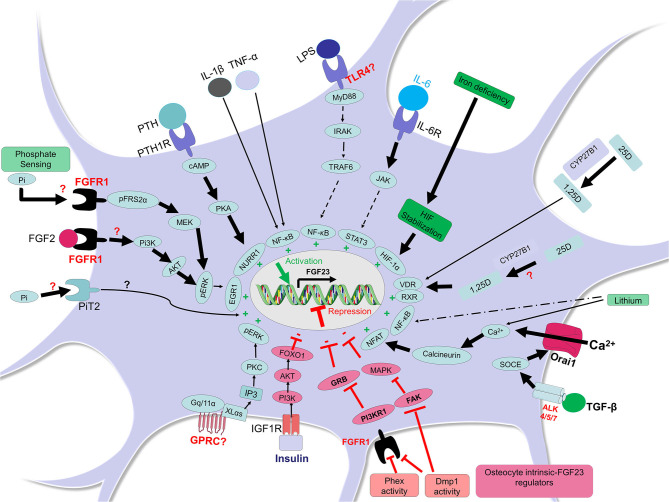
FGF23 regulation in osteocytes. In osteocytes, FGF23 is positively regulated by different factors and pathways (shown in green). In presence of high phosphate, the receptor FGFR1 may sense this condition and the adaptor FRS2α is phosphorylated thus activating FGFR1 and the MEK/pERK/EGR1 pathway leading to FGF23 transcription. It is also possible that the transporter PiT2 senses phosphate increases in osteocyte and responds by inducing FGF23. The activation of FGFR1 by other FGFs such as FGF2 may induce Pi3K/AKT/pERK/EGR1 leading to FGF23 upregulation. The activation of PTH1R by PTH induces cAMP/PKA and the transcription factor NURR1 leading to FGF23 induction. Inflammatory conditions are potent FGF23 inducers; lipopolysaccharide (LPS) may bind to osteocyte toll like receptor 4 (TLR4) and induce MyD88/IRAK/TRAF6/NF-κB to trigger FGF23 transcription (or may act through anemia/hypoxia). The binding of IL-6 to IL-6R in osteocytes may induce JAK/STAT3 and subsequent FGF23 expression. The cytokines IL-1β and TNF-α can induce NF-κB in osteocytes leading to FGF23 transcription. In the condition of iron deficiency, HIF protein is stabilized, an important mechanism involved in FGF23 upregulation. The active form of vitamin D (1,25-dihydroxyvitamin D; 1,25D), which results from the conversion of 25-hydroxyvitamin D (25D) inside or outside the osteocyte, could promote the formation of the complex 1,25D-VDR-RXR which binds the vitamin D receptor element (VDRE, not shown here) and induces FGF23 transcription. The signaling through the TGF-β-Activin Receptors ALK4/5/7 promotes calcium entry in cell through Orai1 and this process activates calcineurin and NFAT and their binding, which induces FGF23. Lithium-induced increased intracellular calcium could also increase FGF23 in osteocytes through calcineurin and NFAT activity. An activation of a yet unknown GPRC could trigger FGF23 transcription through XLαs or Gq/11α and inositol 1,4,5-trisphosphate (IP3) which may activate PKC and stimulate the MAPK pathway. FGF23 can be negatively regulated by other elements and pathways (shown in red). For instance, insulin can bind to IGFR1 and induce PI3K/AKT/FOXO1 pathway which represses FGF23. Phex and Dmp1 activities in osteocytes may control FGFR1/PI3KR1/GRB and cell differentiation to restrain FGF23 production. Dmp1 activity may also neutralize the FAK/MAPK pathway restricting FGF23 expression.

Circulating levels of phosphate control FGF23 production in mammals (25, 26). Although the mechanisms of FGF23 regulation by phosphate are not fully understood, recent studies have implicated the type III sodium phosphate co-transporter PiT2 (Slc20a2) as being required for mediating phosphate-dependent FGF23 production (27). Indeed, *in vivo* studies using dietary protocols in PiT2 knock out mice showed that the deletion of PiT2 results in “inappropriately” normal intact, bioactive FGF23 in the circulation in response to high or low phosphate diet, which should normally increase or decrease FGF23, respectively (27). Using an *ex vivo* system of cultured long bone shafts, parallel studies showed that the PiT2 KO bone shafts failed to undergo Pi-induced FGF23 production, illustrating that PiT2 could be required for FGF23 induction in mouse bone (27). These new findings provided interesting insight underlying the phosphate-dependent regulation of FGF23 secretion, perhaps *via* PiT2 regulating phosphate uptake in osteocytes (27) (Figure 1).

In another study, using proteomic analysis to identify potential upstream sensors in response to elevated phosphate through dietary intervention, the FGFR1 isoform FGFR1c was shown to be activated through the phosphorylation of FGFR substrate 2α (FRS2α, on tyrosine 196) under high phosphate conditions, but in the absence of an FGF ligand. These studies were consistent with the idea that phosphate-sensing mechanisms could control FGF23 production in osteocytes through ligand-independent FGFR1 activation (28, 29). These data suggested that high Pi diet-induced serum FGF23 increases in mice may be mediated by the phosphorylation of FGFR1, a potential “phosphate receptor” upstream of FGF23, which induced the mitogen activated protein kinase (MAPK) and extracellular signal-regulated kinase (ERK) pathways to control FGF23 synthesis (28). Structural and crystallographic studies may be required to understand how an ion can activate an FGFR, as well as whether PiT2 is involved in this regulation (Figure 1).

Additionally, *in vivo* studies have reported that conditional deletion of FGFR1 specifically from the osteocytes of *Hyp* mice, a mouse model of X-linked hypophosphatemia (XLH) that exhibits elevated FGF23 production, improves the rickets and osteomalacia phenotype of these mice in association with a decrease in both FGF23 bone mRNA and circulating protein. These mice also undergo an alleviation of their hypophosphatemic status, as well as an increase of plasma 1,25D levels (30). Furthermore, promoter studies identified that the activation of FGFR1 signaling through FGF2 is dependent on the PI3K-AKT pathway in the MC3T3-E1 osteoblastic cell line, providing evidence that *Fgf23* gene promoter activity is induced by FGFR1 activation (30). In addition to genetic interventions, pharmacologic-based studies have shown that activation of FGFR1 induces FGF23 production and leads to hypophosphatemia, whereas the inhibition of FGFR signaling attenuates FGF23 production (31) (Figure 1).

Another study by Hori et al. investigated whether phosphate could induce reactive oxygen species (ROS) *in vitro*, leading to increased FGF23 expression. Using the osteoblastic cell line UMR-106, these investigators showed that elevated phosphate in the culture media enhances the production of ROS, and that hydrogen peroxide further boosts FGF23 production in a dose-dependent manner, an effect which can be neutralized by an inhibitor of NADPH oxidase (32). These discoveries suggested that *in vitro* phosphate directly enhanced *FGF23* transcription by stimulating NADPH-induced ROS production and the MEK-ERK pathway. How this translates to the signaling in the osteocyte *in vivo* will be an interesting facet to this potential regulatory network.

Circulating FGF23 is markedly elevated during chronic kidney disease (CKD), and this is associated with poor long-term outcomes. By investigating the regulation of FGF23 through FGFR1, Hassan et al. demonstrated that the activation of FGFR1 is essential for the high levels of FGF23 observed during both acute and chronic uremia in mice and rats (33). In addition, in mice treated with the receptor FGFR1 inhibitor PD173074 by oral gavage followed with an acute kidney injury induction using folic acid, the prevailing increased FGF23 was reduced by 50% in calvaria and led to a complete prevention of the circulating intact FGF23 rises (33). In a more prolonged uremic condition using a diet with adenine plus high-phosphorus for 14 days to induce CKD, which resulted in high levels of Fgf23 mRNA and serum FGF23 in rats, an oral gavage intervention with PD173074 given during the last 2 days of treatment reduced FGF23 induction by 75% in calvaria and completely normalized circulating FGF23 (33). Therefore, the FGFRs may play an important role in osteocyte-bloodstream communication to control FGF23 production.

As mentioned above, FGF23 signals *via* the interaction with its receptor FGFR1c and its specific co-receptor Klotho (14, 34). A recent investigation has shown that Klotho could be detected in osteocytes (35) suggesting that FGF23 may be one of the ligands that activates FGFR1 in osteocytes, thereby regulating the *FGF23* gene transcription in a positive feedback loop. The expression of Klotho in osteocytes has been suggested to contribute to bone formation and bone volume increases coupled with enhanced osteoblast activity (35). Although Klotho has been detected in bone (35), the kidney, parathyroid glands and brain remain the primary organs with abundant expression of Klotho to the best of our current knowledge (36–38). However, it is possible that in response to pathological circumstances, bone cells could enhance Klotho expression, thus modulating bone FGFR1 signaling via FGF23.

Through proteolytic cleavage of the membrane-bound klotho (mKL) (39–42), a soluble form of Klotho (sKL) can be liberated into the circulation (43, 44). Soluble Klotho has been described to have a direct role to regulate Wnt and MAPK pathways in osteoblastic UMR-106 cells in concert with the presence of FGF23 (45). Indeed, *in vitro* studies showed that a co-treatment of UMR-106 cells with FGF23 and soluble Klotho activated MAPK signaling, leading to an increase of Dickkopf-1 (DKK1) protein, a soluble inhibitor of Wnt/beta-catenin signaling (45). The induction of Dkk1 through FGF23/FGFR/sKL was shown to be dependent upon the MAPK pathway since the inhibition of this pathway using the MEK inhibitor U0126 completely abrogated p-ERK/ERK induction and abolished downstream Dkk1 expression (45). The binding of the secreted Dkk1 to the receptors Frizzled (Fz) and Lrp5/6 thus promoted the phosphorylation of β-catenin and inactivated the Wnt pathway in osteoblasts (45). Other studies have shown that a treatment of UMR-106 cells with soluble Klotho and FGF23 dose-dependently increased MAPK and Egr1 mRNA responses, an effect which was FGFR- and MEK-dependent, and led to FGF23 upregulation (46). Future studies are needed to confirm potential expression of Klotho in osteocytes and under some circumstances whether FGF23 binding to FGFR1-Klotho complex in osteocytes could induce FGF23 expression.

### Signals Involved in the Positive Regulation of FGF23 by Inflammation and Iron

Inflammation is a complex phenomenon involving multiple immune and non-immune cells which cooperate to respond to endogenous and exogenous events by secreting specific pro-inflammatory and/or anti-inflammatory factors. Pro-inflammatory stimuli such as the cytokines tumor necrosis factor alpha (TNFα), interleukin 1β (IL-1β), the tumor necrosis factor-like weak inducer of apoptosis (TWEAK), and the bacterial component lipopolysaccharide (LPS) have all been shown to dose-dependently upregulate FGF23 in the osteocyte-like cell line IDG-SW3 (47). Particularly, TNF and IL-1β induce FGF23 expression *via* nuclear factor kappa-light-chain-enhancer of activated B cells (NF-κB) (47), a major transcription factor complex involved in the control of cytokines and in the mediation of multiple pro-inflammatory cellular responses. The serine/threonine kinase p38 mitogen-activated protein kinase (p38MAPK), which is activated by several cellular stress stimuli and involved in the transcriptional activity of NF-κB, is another positive regulator of FGF23 in bone cells (48). Other investigations have shown that the serine/threonine kinase protein kinase C (PKC), which drives FGF23 expression in response to phorbol ester 12-O-tetradecanoylphorbol-13-acetate (PMA), can be suppressed by 75% in the presence of the PKCα/β inhibitor Go6976. These studies suggested that PKC is a positive regulator of FGF23 synthesis in IDG-SW3 osteocytic cells *via* NF-κB (49). Furthermore, Notch signaling, which can be mediated by pro-inflammatory cytokines such as TNF-α (50), has been described by Tamamura et al., to positively regulate FGF23 expression which colocalizes with Notch signaling downstream targets, Notch, and Hes1 in mouse osteocytes and UMR-106 osteoblastic cells (51). A study also identified a potential new pathway mediated by G protein–IP3/PKC events to control FGF23 production (52). It was shown that the ablation of the extra-large Gα subunit (XLαs) or Gq/11α in osteocyte-like Ocy454 cells activated IP3/PKC and stimulated the MAPK/ERK1/2 pathway leading to FGF23 induction (52). It is assumed that the trigger of the IP3/PKC/MAPK pathway is controlled by a G protein-coupled receptor which remains to be identified (Figure 1).

More recently, the use of CRISPR/Cas9 technology has helped to identify control regions for the *Fgf23* gene in response to various inflammatory stimuli in mice. An enhancer site − 16kb from the transcription start site of the murine *Fgf23* gene was deleted via CRISPR, and this study demonstrated that FGF23 expression in bone, bone marrow, spleen, liver, thymus, and intestine was attenuated in response to LPS injection. This deletion was also associated with decreased intact FGF23 compared to normal mice treated with LPS. Similar effects were observed in response to direct TNF-α and IL-1β injections, highlighting the importance of this genomic region in mediating inflammation-induced FGF23 expression (53). Further studies showed that an additional proximal enhancer region in *Fgf23* mediates LPS-induced FGF23 expression *in vivo* through binding motifs to several known transcription factors that mediate inflammation, including NF-κB (54).

Persistent iron deficiency in mice has been shown to induce a pro-inflammatory state (55) that induces a heightened inflammatory response to stimuli such as LPS (55, 56). Both iron deficiency and inflammation increase *FGF23* transcription by activating among other signaling pathways, Hif1α, and associated MAPK signaling, in osteocytes (57, 58). Wild type mice fed an iron deficient diet for 8 and 12 weeks showed 5- and 10-fold increases of Fgf23 transcripts, respectively, in femur/tibia samples (57). *In vitro*, treatment of UMR-106 cells with the iron chelator, deferoxamine (DFO) resulted in marked increases in Fgf23 mRNA, partially dependent on MAPK activity in association with Hif1α stabilization (57, 58) (Figure 1). These discoveries suggest that absolute iron deficiency, characterized by a depletion of both iron stores and circulating iron, promotes *FGF23* transcription through Hif1α. Similarly, a functional iron deficiency *via* a treatment of mice with hepcidin also increased bone Fgf23 mRNA expression (58).

The cytokine IL-6, a known marker of inflammatory states has also been shown to contribute to FGF23 production. In a mouse model of folic acid–induced acute kidney injury, bone Fgf23 mRNA expression increased together with serum FGF23 as well as several circulating cytokines including IL-6 (33). When fed with an adenine diet to induce CKD, IL-6 knock-out mice failed to increase bone Fgf23 mRNA, resulting in an attenuation of circulating FGF23 levels in comparison to wild-type mice with CKD; these data suggest a direct contribution of IL-6 to the increased FGF23 observed during CKD (59). Further, *ex vivo* and *in vitro* studies have shown that a treatment of calvaria organ cultures and UMR-106 cells with the IL-6/soluble IL-6 receptor fusion protein induced STAT3 phosphorylation, and increased *Fgf23* promoter activity, suggesting a direct effect of IL-6 on the positive regulation of FGF23 expression (59).

### Hypoxia and Erythropoietin as Positive Regulators of FGF23

Hypoxia inducible factors (HIFs) are constitutively expressed transcription factors that are critical for sensing oxygen and iron levels in the body. Oxygen and iron are important co-factors for the function of HIF-prolyl hydroxylase domain enzymes (HIF-PHDs), which help maintain the constant turnover of HIFs by marking them for degradation. As stated above, it was shown that iron deficiency can drive FGF23 expression in a mouse model of autosomal dominant hypophosphatemic rickets (ADHR) (57). Wild type and ADHR mice (harboring the FGF23-R176Q stabilizing ADHR mutation) fed an iron-deficient diet had elevations in “total” serum FGF23 (intact bioactive FGF23 + proteolytic fragments), however only iron-deficient ADHR mice had increases in intact FGF23 due to their inability to cleave and inactivate the bioactive form of FGF23. This and subsequent studies helped tie together iron handling and phosphate homeostasis, a link that can likely be explained through HIF activity. An iron-deficient state leads to HIF stabilization due to the lack of iron as a co-factor for the HIF-PHDs to tag HIF for degradation. *In vitro* studies have shown HIF-mediated induction of FGF23 mRNA expression in two different osteoblast cell lines, as well as evidence of HIF binding to the FGF23 promoter (60). More recently, a prospective study in ADHR patients that co-presented with an iron deficiency phenotype demonstrated that iron repletion in these patients reduced their circulating levels of FGF23 and normalized serum phosphate levels. Thus, providing oral iron is a novel therapeutic approach to this disease (61). This finding was supported by studies in iron-deficient women showing that rescue of iron deficiency using iron dextran lowered the prevailing elevated FGF23 (62). It is important to note the type of intravenous iron used, as it has been well-documented that certain types of iron formulations, especially those with carboxymaltose backbones can cause hypophosphatemia due to elevating intact FGF23 (63), potentially through altering FGF23 intracellular proteolysis (see below).

Erythropoietin (EPO) is a hormone made in the kidney that induces new red blood cell production in the bone marrow in response to anemia or blood loss. Recombinant human EPO (rhEPO) is currently used as a therapy to treat anemia related to CKD. Recently, several studies revealed that this hormone can induce FGF23 expression (64–66). WT mice given acute rhEPO treatment after 6 h or after 3–4 days of consecutive injections resulted in elevated total and intact FGF23 and bone marrow Fgf23 mRNA expression (64). To understand the effects of chronically elevated EPO, reports using the transgenic Tg6 mouse model of EPO overexpression showed that these mice have basal elevations in total and intact FGF23 (65, 67). Chronic overexpression of EPO can lead to iron deficiency, so to determine whether the iron deficiency was causing the elevated FGF23, mice were given iron dextran. This only modestly decreased intact FGF23, showing that EPO was primarily driving FGF23 expression (65). In healthy human subjects, total FGF23 was elevated 24 h after a single rhEPO dose with no changes in intact FGF23 (66). In another study, a small population of anemic patients with normal kidney function were given a single dose of rhEPO. This increased total and intact FGF23 over the span of 12–18 h after the injection (64). Few *in vivo* studies have examined the effect of curing anemia of CKD on mineral metabolism. Most recently, a single rhEPO injection in CKD mice did increase total serum FGF23 after 6 and 24 h but had no effect on intact FGF23 (65).

To mitigate potential adverse effects of high rhEPO treatment, new strategies are leveraging the HIF system by creating inhibitors of the HIF prolyl hydroxylases (HIF-PHDs) called HIF-PHDs inhibitors (HIF-PHI). These therapeutics have become increasingly important for anemic patients with CKD, where they promote endogenous EPO production by stabilizing HIFs. This class of drug also reduce hepcidin levels to increase iron utilization in tissues thereby creating a synergistic effect in providing iron availability to newly forming red blood cells (68). Recent studies have shown in normal mice that several HIF-PHIs can regulate Fgf23 expression. For instance, FG-4592 (Roxadustat) was shown to increase bone Fgf23 mRNA and circulating intact FGF23 (58, 66). Elevations in total and intact FGF23 were observed in WT mice treated with the HIF-PHI BAY 85-3934 (Molidustat) after 6 h that returned to baseline by 24 h. This study showed that elevated EPO precedes increases in FGF23 in response to HIF-PHI BAY85-3934, suggesting that the cause for elevated FGF23 in response to this treatment was due to elevated EPO. This was confirmed by treating HIF-PHI mice with an EPO-neutralizing antibody, which completely attenuated the increased serum total FGF23 (69). A recent paper, however, showed that EPO and HIF-PHI treatment of anemic mice with CKD resulted in suppressed FGF23. This study suggested that under conditions of anemia, as opposed to mice with normal iron homeostasis, rescuing iron utilization during CKD may be a more potent suppressor of FGF23 than EPO and HIF-PHIs are stimulators (70). Although EPO and HIF-PHI can induce FGF23, further studies are required to delineate the mechanisms directing these components on osteocytic FGF23 production.

### Signals Involved in the Transcriptional Regulation of FGF23 by PTH

Parathyroid hormone (PTH) is a hormone secreted by the parathyroid glands that regulates calcium utilization through its effects on bone, kidney, and intestine (71–73). During CKD, a secondary hyperparathyroidism occurs in which PTH is excessively secreted, in response to factors such as hyperphosphatemia, hypocalcemia, and low 1,25D levels, to potentially promote elevated FGF23 (74). Using bioinformatics and chromatin immunoprecipitation assays, studies have reported that Nurr1 is an essential transcription factor involved in FGF23 upregulation in response to PTH in UMR-106 cells. Furthermore, in a mouse model of CKD, the administration of a calcimimetic, which is known to activate the calcium-sensing receptor in different tissues (75) with the goal to attenuate PTH levels and actions, reduced FGF23 concentrations as well as bone Nurr1 mRNA and protein levels (76). To test the relationships between PTH and FGF23, a mouse model with constitutive activation of PTH receptor (PTHR) signaling in osteocytes was used by Rhee et al. in a report that showed that PTHR activation increased FGF23 expression *in vivo* and *in vitro* through cAMP and Wnt-dependent mechanisms (77). In addition, Knab et al. showed that PTH-induced increases in FGF23 expression were PKA-dependent in osteocyte-like cells, suggesting that FGF23 production is regulated by the cAMP/PKA/Nurr1 pathway in response to PTH (78) (Figure 1).

### Signals Involved in the Positive Regulation of FGF23 by TGF-β, Calcineurin, and NFAT

TGF-β has been reported to regulate the extracellular matrix by activating ROS, which increases calcium influx and activates calcineurin (79–81). Using UMR-106 cells, TGF-β2 has been described to enhance store-operated Ca^2+^ entry (SOCE) and induce the stimulation of FGF23, an effect significantly attenuated by both the inhibitor of TGF-β type I receptor activin receptor-like kinases (ALK5, ALK4, and ALK7) SB431542 and SOCE inhibitor 2-APB (82). Recent investigation has shown that the synthesis of FGF23 in UMR-106 cells can be induced by SOCE through Orai1 (83). In addition, the Ca^2+^ entry activates the phosphatase calcineurin, which dephosphorylates nuclear factor of activated T cells (NFAT) thereby stimulating its transcriptional activity and targeting the *Fgf23* gene. This suggested that FGF23 may be positively regulated by calcineurin-NFAT signaling (84). Further analyses confirmed that either the inhibition of calcineurin using ciclosporin A (CsA) and tacrolimus (FK-506) or the blocking of the interaction between calcineurin and NFAT using the inhibitor INCA-6 reduced the abundance of *Fgf23* transcripts as well as FGF23 protein (84). Additionally, lithium, a widely used drug for the treatment of mood disorders and known to modify Ca^2+^ signaling, stimulated the release of FGF23, partially through NF-κB dependent up-regulation of Orai1 transcription and SOCE in UMR-106 cells (85) (Figure 1).

### Signals Involved in the Positive Transcriptional Regulation of FGF23 by Calcitriol

Early FGF23 physiological studies demonstrated that the administration of 1,25D dose-dependently increased both serum FGF23 as well as serum inorganic phosphorus in normal rats (25). In wild type mice with normal renal function, injection of calcitriol increased serum FGF23 levels 1-week post treatment by 15-fold. Calcidiol (25-hydroxyvitamin D), although with a lesser effect could also induce FGF23 in normal mice (86). In a mouse model of adenine diet-induced CKD, a 5 weeks-regimen induced a 40-fold increase of circulating FGF23. However, in the background of a global deletion of Cyp27b1, the gene encoding the enzyme vitamin D 1-α-hydroxylase involved in the formation of calcitriol (1(OH)ase^−/−^ mice), only a 2-fold circulating FGF23 was observed with this treatment, suggesting a contribution of calcitriol to the increased FGF23. At the bone compartment level, a specific deletion of Cyp27b1 in osteoblasts reduced FGF23 induction in long bones from 58-fold in normal mice treated with a 5-weeks adenine diet to a 10-fold induction, suggesting a potential role of a local osteoblastic vitamin D conversion process in the induction of bone FGF23. This attenuation of increased FGF23 was independent of a potential reduction in PTH levels since plasma PTH remained elevated in response to the adenine diet-induced CKD in the mice with global deletion of Cyp27b1, as well as those with osteoblast-specific deletion of Cyp27b1 (86) (Figure 1).

## Negative Regulators of FGF23

### Dentin Matrix Acidic Phosphoprotein 1 and Phosphate-Regulating Gene With Homologies to Endopeptidases on the X Chromosome

The disease XLH is characterized by hypophosphatemia and impaired mineralization caused by mutations of the *phosphate-regulating gene with homologies to endopeptidases on the X chromosome* (PHEX). Loss of PHEX leads to the overproduction of FGF23 in osteocytes, causing hypophosphatemia with bone mineralization impairment, and thus bone fragility. Similar to XLH, recessive loss-of-function mutations in the dentin matrix protein-1 (DMP1) gene, a member of small integrin-binding ligand N-linked glycoprotein (SIBLING) proteins, is responsible for a human phosphate wasting and impaired bone mineralization disease termed autosomal recessive hypophosphatemic rickets type 1 (ARHR1). It was shown that a lack of DMP1 in both humans and mice markedly increased FGF23 expression in bone (87).

To gain insight into the mechanisms by which PHEX mutations upregulate FGF23 expression, studies have been designed to investigate the local effects in bone from a mouse model of XLH (Hyp mice) in a normal hormonal environment in comparison to the function of wild type bone in the abnormal metabolic environment of Hyp mice. Using a surgical procedure to perform intramuscular bone cross-transplantations between wild-type and Hyp mice, a study found that increased FGF23 expression in Hyp bone results from intrinsic PHEX deficiency from bone, since FGF23 was increased in Hyp osteocytes before and after explantation into WT mice but was not increased in WT osteocytes after explantation into Hyp mice. This evidence suggested that the mechanisms whereby PHEX mutations lead to increased FGF23 expression in osteocytes is intrinsic to bone (88).

Similar to the phenotype resulting from PHEX inactivation, the inactivation of *Dmp1* in mice resulted in equivalent intrinsic bone mineralization defects associated with increased FGF23 expression in osteocytes (89–91). Using cortical bone isolated from 12-days old WT, Hyp, Dmp1^−/−^, and Hyp/Dmp1^−/−^ mice to perform a genome-wide microarray analysis, the phosphatidylinositol 3-kinase regulatory α subunit (PIK3R1) and growth factor receptor-bound protein 2 (GRB2) pathways were identified as potential common signaling controlled by PHEX and DMP1 to regulate *FGF23* promoter activity through FGFs/FGFR in osteocytes (91, 92) (Figure 1). These findings highlight that the activation of FGFRs which contribute to FGF23 production in osteocytes may be independent from the phosphate sensing pathways described above. Additionally, recent *in vivo* and *in vitro* studies suggested a direct negative regulation of DMP1 in FGF23 expression in osteocytes by activating FAK-mediated MAPK signaling, thus coordinating the extracellular environment of osteocytic lacunae as well as bone metabolism (93) (Figure 1). The creation of the floxed-Fgf23 mouse has recently emerged as a critical tool to understand FGF23 function *in vivo* (94). To this end, flox-Fgf23 mice were mated to the global eIIa-cre which mimicked the phenotype of the Fgf23-KO mouse. Fgf23 was also specifically deleted from either the osteoblast lineage using the Col2.3 promoter to drive Cre expression, or from late osteoblasts/osteocytes using the Dmp1-Cre. This resulted in ~50% reduction of iFGF23, with compromised ability to respond to changes in phosphate (94), demonstrating the specificity of osteoblast/osteocyte FGF23 production in response to metabolic changes.

### Negative Regulation of FGF23 by Insulin and Insulin-Like Growth Factor 1

Recent studies reported insulin and insulin-like growth factor 1 (IGF1) as negative regulators of FGF23 production *in vitro* as well as in mice and humans (95). *In vitro*, insulin and IGF1 down-regulated FGF23 production by inhibiting the transcription factor forkhead box protein O1 (FOXO1) through phosphoinositide 3-kinase (PI3K)/protein kinase B (PKB)/Akt signaling in UMR-106 cells (95). *In vivo*, insulin deficiency resulted in an increase of serum FGF23 concentrations in mice, which was reversed by insulin administration. Interestingly, in women subjects, an increase in plasma insulin levels following an oral glucose administration correlated negatively with plasma FGF23 concentrations (95) (Figure 1).

## FGF23 Cleavage: A Physiological and Endogenous Mechanism to Attenuate FGF23 Bioactivity

**Figure 2:**
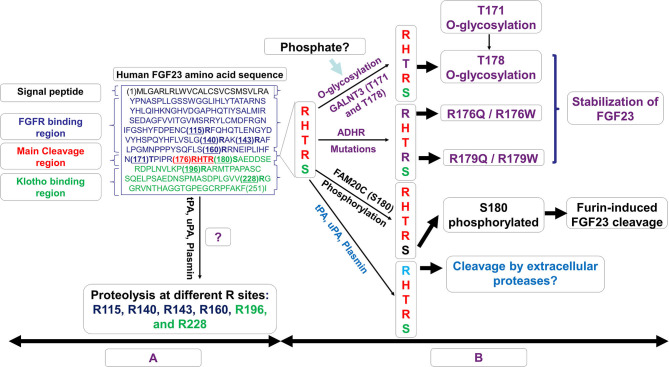
Schematic representation of the possible fates of FGF23. In **(A)**, the amino acid sequence of human FGF23 is shown with its signal peptide, FGFR binding region, the RXXR motif/cleavage sequence, and the Klotho binding region. In **(B)**, the possible fates of mature FGF23 after the loss of its signal peptide sequence is displayed. The *O*-glycosylation of T_178_, a process controlled by N-acetylgalactosaminyltransferase 3 (GALNT3) and which requires a precedent glycosylation at T_171_, stabilizes and prevents FGF23 cleavage. This process may also be stimulated by increased levels of phosphate which induces GALNT3 and the glycosylation. Genetic modifications such as autosomal dominant hypophosphataemic rickets (ADHR) mutations R176Q, R176W, R179Q, and R179W stabilize FGF23 and counteract its natural cleavage. Phosphorylation at position S_180_ by FAM20C increases FGF23 cleavage controlled by the furin/subtilisin-like proprotein convertase. FGF23 proteolysis may occur at R_176_ by extracellular proteases of the plasminogen activation system, tissue-type PA (tPA), urokinase-type PA (uPA), and plasmin. This proteolysis could potentially occur at the RXXR motif (R176) as well as at different arginine residue sites at R_115_, R_140_, R_143_, R_160_, R_196_, and R_228_.

FGF23 is synthesized as a 251-amino acid protein, primarily in osteocytes. The FGF23 signal peptide is represented by the first *N*-terminal 24 aa. The cleavage of the signal peptide results in a release of a mature peptide that can be secreted as the bioactive hormone, referred to as “intact” FGF23 (“iFGF23”). To control the bioactivity of iFGF23, the protein can be cleaved at the subtilisin-like proprotein convertase (SPC) site R_176_HTR_179_/S_180_AE (RXXR/SAE motif) generating at least two fragments identified as an *N*-terminal fragment which is structurally similar to other FGF family members and a more unique *C*-terminal tail (21, 96). The proteolytic cleavage of excess iFGF23 represents a critical secondary regulatory mechanism to maintain stable serum iFGF23 and normal serum phosphate. The absence of this proteolytic activity in humans through the *FGF23* gene mutations R176Q, R176W, R179Q, and R179W are causative for autosomal dominant hypophosphatemic rickets (ADHR), characterized by elevated intact FGF23 and hypophosphatemia (21) (Figure 2). In mice carrying the ADHR point mutation R176Q-Fgf23, in response to absolute iron deficiency using dietary intervention, the iFGF23 levels were elevated due to the stabilization of the bioactive FGF23 (57). This elevated iFGF23 condition in response to iron-deficient diet in ADHR mice caused similar phenotypes as observed in ADHR/XLH patients, such as alterations in genes controlling phosphate reabsorption and 1,25D production, and a hypophosphatemic bone disease (57). These findings suggested that iron status is a synergistic factor of the ADHR phenotype, and that ADHR is a disease of gene-environment interactions (57).

The cleavage of FGF23 is controlled by the serine endoprotease furin, a subtilisin-like convertase, at the site R_179_/S_180_ in response to several stimuli including PTH (78). Previous investigations have shown that an iron deficient state also promotes iFGF23 cleavage leading to increased secretion of FGF23 fragments but normal iFGF23 in WT mice (57, 58). In HeLa Cells, it was described that an iron deficient state induces furin upregulation *via* the stabilization of Hif1α (97), a similar mechanism which occurs in osteocytes. Posttranslational modifications of iFGF23 can occur via the *O*-glycosylation of Thr_178_ in the furin proprotein processing motif RHT_178_R_179_, which stabilized FGF23 (98) (Figure 2). This glycosylation process at Thr_178_ is controlled by the enzymatic actions of N-acetylgalactosaminyltransferase 3 (GALNT3) which can be upregulated under high phosphate conditions potentially *via* the control of FGFR1c activation and the induction of the transcriptional activators early growth response 1 (EGR1) and ETS variant 5 (ETV5) (28).

In a recent study, Thr_178_ was identified as a poor substrate site with limited glycosylation acceptance, likely a protective mechanism to prevent cellular resistances to FGF23 cleavage. Interestingly, Thr_178_ glycosylation was shown to require a previous glycosylation at Thr_171_ before generating a furin-resistant and secreted stable iFGF23 (99) (Figure 2). These new discoveries suggest that GALNT3 specificity for FGF23 and its ability to control circulating levels of bioactive FGF23 is a control point achieved by FGF23 being a rather poor substrate for this enzyme (99). In contrast to the *O*-glycosylation induced by GALNT3 which stabilizes FGF23, the phosphorylation at position S_180_ by the kinase FAM20C inhibited *O*-glycosylation of FGF23, thus promoting FGF23 cleavage (98) (Figure 2). Indeed, recessive inactivating mutations in human *FAM20C* cause ARHR type 3 (ARHR3; Raines syndrome), consistent with its role as an FGF23 de-stabilizer (100, 101).

Besides furin which is thought to cleave FGF23 mainly intracellularly, the extracellular proteases of the plasminogen activation system, tissue-type PA (tPA), and urokinase-type PA (uPA), as well as plasmin may also display proteolytic activity on FGF23 protein at the RXXR motif (R_176_), with potential additional cleavages at arginine residues R_114_, R_140_, R_143_, R_160_, R_196_, and R_228_ (102). Of note, Klotho knock out mice as well as mice with acute kidney injury, which both exhibit elevated levels of intact FGF23, also display elevated plasminogen activator inhibitor-1 (103, 104). Thus, the proteolysis of FGF23 by tPA, uPA, and plasmin may potentially regulate the levels of active FGF23 thus in part, controlling phosphate and mineral homeostasis.

These collective studies demonstrated that FGF23 protein cleavage is a dynamic process which can be adjusted after production at the cell level with phosphorylation and glycosylation dictating the levels of bioactive FGF23 depending upon the osteocyte cell state. Certainly, future studies are needed to understand the regulation of GALNT3, furin, FAM20C, and potentially the enzymes of the plasminogen activation system in the coordinated control of FGF23 production and bioactivity.

## FGF23 Renal Signal Transduction

In contrast to paracrine FGFs, such as FGF1 and FGF2, endocrine FGFs such as FGF23 lack the heparin-binding domain in their *C*-terminus, which enables escape from the osteocyte cell matrix after secretion and their actions on distant target organs including kidney (14, 105). FGF23 acts primarily on kidney to promote phosphaturia and parallel reductions in 1,25D (14, 34). The phosphaturic activity of FGF23 is critical in CKD, preventing and delaying hyperphosphatemia and vascular calcifications as FGF23 progressively rises with the loss of renal function (106). In a study among patients undergoing hemodialysis, high serum phosphate levels across a quartile of patients (>5.5 mg/dl) was associated with a 20% increase in the multivariable adjusted risk of death, as compared with normal levels (3.5–4.5 mg/dl) (107). These findings underscore the importance of controlling circulating phosphate in kidney disease patients. During CKD, as kidney function decreases with a progressive decrease of glomerular filtration rate (GFR), the kidney loses functioning nephrons decreasing the overall excretion capability of the kidney. The dramatic rise of FGF23 during CKD is likely to attempt to boost the excretory capacity of the existing nephrons to maintain normal serum phosphate levels. This is likely the primary reason why early stage CKD patients exhibit normal serum phosphate over much of the disease course [for review (108)].

**Figure 3:**
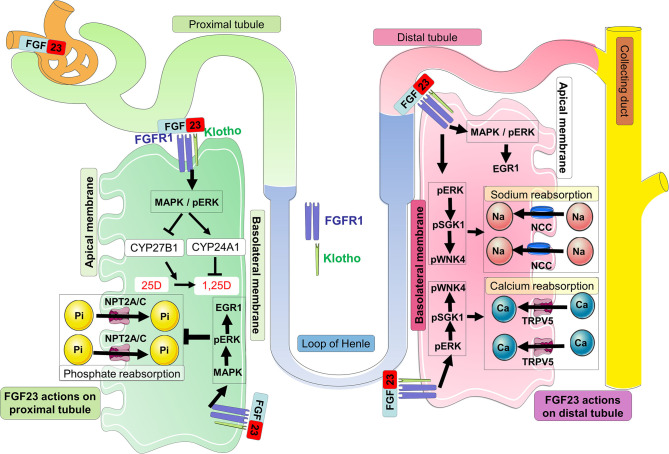
FGF23 signaling in the kidney. In kidney proximal tubules, circulating FGF23 binds to FGFR1-Klotho complexes at the basolateral membrane, and activates the MAPK signaling cascade involving ERK1/2. This signaling leads to the internalization and degradation of NPT2A/C and the decrease of urinary phosphate reabsorption promoting phosphate wasting. In the proximal tubule, FGF23 signaling induces downstream mechanisms which suppress the transcription of the vitamin D 1α-hydroxylase (CYP27B1) and increases the transcription of the vitamin D 24-hydroxylase (CYP24A1); both events work in concert to limit the conversion of 25D to 1,25D as well as degrading 1,25D into inactive metabolites. In the kidney distal tubule, circulating FGF23 binds to the FGFR-Klotho receptor complex at the basolateral membrane, and activates the MAPK/pERK/EGR1 pathway as well as cascade signaling for ERK1/2, SGK1, and the WNK4 complex via their phosphorylation. Activation of WNK signaling increases the expression of TRPV5 and NCC at the apical membrane promoting the reabsorption of calcium and sodium, respectively.

FGF23 actions are likely mediated by FGF receptors FGFR1c, FGFR3c, and FGFR4 and the co-receptor Klotho, a single-pass transmembrane protein highly expressed in kidney (109–113). FGF23 binds to FGFR1c and the interaction between these two proteins with the co-receptor Klotho (which dramatically increases the binding affinity of the complex FGF23-FGFR-Klotho) triggers potent FGF23 signaling and activity (114). In kidney, FGF23 signaling on the basolateral side of proximal nephron cells causes the internalization of the sodium-dependent phosphate co-transporters NPT2A and NPT2C from the apical surface. These actions decrease phosphate reabsorption processes from the kidney (115) (Figure 3). A global genetic deletion of FGF23 in mice resulted in severe hyperphosphatemia, due to the absence of the FGF23-mediated phosphaturia mechanism (116). In addition, FGF23 signaling regulates vitamin D metabolism *via* the modulation of the vitamin D metabolic enzymes expression as well as regulating calcium and sodium reabsorption processes (Figure 3).

### Unbiased Approaches to Understanding FGF23 Function in Kidney

Unbiased studies using large-scale microarray and gene expression approaches from FGF23 transgenic (FGF23 Tg) mice and WT littermates have identified several renal genes associated with increased FGF23. Particularly, genes involved in the phosphate reabsorption process such as Npt2a, Pdzk1 (a scaffolding protein known to interact with Npt2a) and Klotho, the co-receptor for FGF23 were among the genes which showed significantly decreased expression (117). The angiotensin I converting enzyme 2 (Ace2), known to decrease Angiotensin II with potential disruption of the renin-angiotensin system balance (118) was also decreased in FGF23 Tg kidney (117). It was later hypothesized that FGF23 induced the activation of FGFR1, leading to Ace2 reduction, causing hypertension (119). Other genes such as Atp1a2, which has been described to interact with Klotho and regulate calcium metabolism (120), was 2.5-fold- upregulated (117) and lipocalin 2, a neutrophil-derived marker previously described to be involved in CKD progression in mice and humans (121), was also modestly induced (117).

Other approaches using an acute 1-h FGF23 injection revealed that the MAPK related transcription factor Egr1 is typically the most up-regulated gene in microarray datasets testing FGF23 bioactivity. It was also determined that EGR1 binding near genes encoding Npt2a, Npt2c, as well as scaffold proteins (NHERF1-3, EZR and GABARAP), and trafficking proteins (megalin and vacuolar ATPase), known to putatively mediate NPT2A protein internalization and degradation, were increased after FGF23 injection. Collectively, these results suggested a coordinated regulation of renal Pi transport genes through EGR1 (122).

### EGR1: An FGF23 Biomarker and Beyond

As mentioned above, it has been well-studied that FGF23 delivery induces EGR1, a transcription factor and downstream marker of MAPK signaling, in mice and cells (112, 123). It was shown in the Hyp mouse that MAPK/ERK1/2 signaling is activated in the kidney and this pathway induction drives EGR1 (112, 123). Other studies revealed that an inhibition of ERK1/2 signaling using a selective MEK inhibitor, PD0325901, was associated with a reduction of renal Egr1 upregulation induced by a Phex deletion. This led to improvement in the hypophosphatemia associated with decreased NPT2a protein expression, correction of 1,25D and calcium deficiency, with a reduction of cortical porosity (124, 125).

To further understand the contribution of Egr1 on FGF23-dependent regulation of renal Pi and vitamin D metabolism, the Egr1 knockout mouse (Egr1^−/−^) has been studied with interesting findings. Reports showed that the effect of FGF23-induced decreases in renal Npt2a and Npt2c proteins is completely abrogated in Egr1^−/−^ mice (122). To potentially attenuate Egr1 activity, FGF23 also upregulates two isoforms of Nab2 which are known to be corepressors of Egr1 (126); however whether this is a direct or an indirect mechanism through Egr1, which is known to induce Nab2 thus establishing a negative feedback loop (127), remains to be investigated.

### Physiological Role of Renal FGF23 Receptors

Four FGFR isoforms (FGFR 1-4) have been described in mammals and an alternative splicing of these isoforms can generate seven major FGFR proteins (FGFRs 1b, 1c, 2b, 2c, 3b, 3c, and 4) (105). FGFR1, FGFR3, and FGFR4 are the most expressed FGFRs in the mouse kidney with potential functional roles in mineral homeostasis (128–130). In mice, a deletion of Fgfr1, Fgfr2, Fgfr3, and Fgfr4 from renal proximal tubules induces hyperphosphatemia, hypercalcemia, hypervitaminosis with elevated FGF23 (128). Other studies using mice with a specific deletion of FGFR1 in distal tubule segments have suggested that FGF23 activates FGFR1/alpha-Klotho complexes in the distal tubule leading to an increase of sodium-chloride symporter (NCC)-dependent sodium (Na) reabsorption, a decrease of Ace2 and renal KIotho, leading to increased blood pressure and hypertension (119). Further, activation of FGFR1 using pharmacologic intervention normalized blood pressure in Hyp mice, a mouse model of elevated FGF23 (119). Although the roles of FGFR3 and FGFR4 in kidney are not completely understood, *in vivo* studies proposed that FGFR3 and FGFR4 may contribute by acting in concert with FGFR1 to mediate FGF23 effects in kidney. Although their roles may be less substantial at the physiological level, under conditions of high circulating FGF23 in Hyp mice, the deletion of FGFR3 leads to a feedback stimulation of Fgf23 mRNA expression in bone (129, 131), suggesting complex kidney-bone crosstalk.

The co-receptor KL is required for high-affinity FGF23 activity in the kidney. Whole-nephron deletion of Klotho in mice results in renal FGF23 resistance, characterized by hypervitaminosis D, hyperphosphatemia, and other phenotypes that may resemble premature aging (39, 132). However, provision of a low phosphate diet to KL-null mice reversed the severe phenotypes, showing that the majority of KL-null phenotypes are due to extreme phosphate imbalances (133, 134). Klotho is highly expressed in the distal tubule segments of the nephron in comparison to its relatively modest expression in proximal tubules (123) although the phosphate reabsorption occurs primarily in the proximal tubules due to an abundant expression of the sodium phosphate transporters Npt2a and Npt2c (135–137). To delineate and identify the main effector sites of FGF23 actions in kidney, recent elegant studies have been performed using mouse models with nephron segment-specific deletion of Klotho in concert with a full characterization of mineral metabolism of these mice (138, 139). Olauson et al. generated a mouse model with deletion of Klotho in distal tubular segments (Ksp-KL2/2) which was characterized as fertile with a normal gross phenotype despite a disrupted mineral metabolism. These phenotypes were in contrast to Klotho-null mice which are not fertile in addition to undergoing premature death and severe vascular calcifications (138). By using immunohistochemistry analysis, investigators showed that partial deletion of Klotho in distal tubule resulted in hyperphosphatemia with elevated plasma FGF23 and increased Npt2a protein expression in the proximal tubule apical membrane (138). In other studies where Klotho was conditionally deleted from renal proximal tubule, the mineral metabolism phenotype was variable depending upon the strategies used to perform specific Klotho deletion. Indeed, in the studies of Ide et al., only a mild phenotype on mineral metabolism with a decrease of urinary phosphate was observed when Klotho was deleted from proximal tubules using three different proximal tubule specific *Cre* transgenic mice: Kap-Cre (kidney androgen-regulated protein), Slc34a1-Cre (sodium phosphate cotransporter-2a1) or Pepck-Cre (phosphoenolpyruvate carboxykinase) (139). The latter, harbors elevated serum iFGF23 and a slight increase in Npt2a protein (139). In contrast, other studies using an inducible promoter-Cre, Ndrg1-Cre, to delete Klotho from proximal tubules revealed a more pronounced effect on mineral metabolism with markedly elevated iFGF23 and hyperphosphatemia (128). The phenotypic differences across these mouse models could be explained by the variability in Cre-mediated recombination efficiency, which can be factored by the cell-type specificity of Cre expression, the Cre expression efficiency in specific cell types, and the recombination feasibility from different genetic modification strategies.

It was later shown that FGF23 signaling can cross-talk with PTH signaling to control mineral metabolism. A deletion of both PTH1R and Klotho from the kidney proximal tubule (PT-PTH1R/KL^−/−^ mice) led to a severe disturbance of mineral metabolism including hyperphosphatemia at baseline and increased circulating phosphate in response to high phosphate diet (140). The hyperphosphatemia observed in PT-PTH1RKL^−/−^ mice was associated with elevated circulating FGF23, PTH, decreased circulating 1,25D and increased Npt2a (140). These new data underscore that FGF23 and PTH signaling pathways can interact in kidney thus coordinating renal phosphate handling in the proximal tubule (140, 141).

Using animal models, studies confirmed over recent years that FGF23 is a negative regulator of 1,25D production. Indeed, FGF23 potently inhibits the expression of the 25(OH)D-1α-hydroxylase *CYP27B1* in the renal proximal tubule while stimulating *in contrario* the expression of the vitamin D catabolic enzyme *CYP24A1* at the mRNA level (23, 142–144). Mice with global deletion of the Fgf23 gene displayed elevations in serum 1,25D by 2-4 fold (144). Since FGF23 acts as a requisite partner with its co-receptor Klotho to control mineral metabolism, *Klotho* ablation in mice resulted in a strikingly similar phenotype to the *Fgf23*-null mice, including increased serum levels of 1,25D associated with increased renal Cyp27b1 expression (39, 145). Interestingly, the premature aging-like phenotype of *Fgf23*^−/−^ and *Klotho*^−/−^ mice can be completely rescued using a genetic approach to ablate 1,25D synthesis through the generation of double mutant *Fgf23*^−/−^/*1*α*(OH)ase*^−/−^ and *Klotho*^−/−^/*1*α*(OH)ase*^−/−^ mice (146, 147). These data suggested that increased vitamin D played a major role in the abnormal mineral ion metabolism and soft-tissue anomalies observed in *Fgf23*^−/−^ and *Klotho*^−/−^ mice. Although Klotho deletion results in hypervitaminosis, and the kidney is the predominant organ expressing Klotho, studies using targeted deletion of Klotho in the proximal or distal tubule segment of the nephron have shown an overall modest effects on circulating vitamin D levels (138, 139), likely due to endocrine compensation.

It has also been described that the deletion of vitamin D receptor (VDR) in *Fgf23*^−/−^ and *Klotho*^−/−^ mice rescued these mice from an early lethality phenotype due to the absence of vitamin D signaling causing reduced phosphate absorption (148, 149). Therefore, *Fgf23*^−/−^/VDR^Δ/Δ^ and *Kl*^−/−^/VDR^Δ/Δ^ double mutant mice can be used to examine the roles of FGF23 and Klotho at older ages by keeping these mice on a rescue diet rich in calcium, phosphorus, and lactose (150, 151) with the goal of preventing hypocalcemia and severe hyperparathyroidism due to the non-functioning VDR status.

Study of the *Fgf23*^−/−^/VDR^Δ/Δ^ and *Kl*^−/−^/VDR^Δ/Δ^ mice showed that the deletion of *Fgf23* or *Klotho* leads to a decrease in the membrane abundance of NCC (the sodium chloride cotransporter) in the kidney distal tubule and subsequently to decreased Na^+^ reabsorption (152). In contrast, treatment of WT mice with FGF23 over 5 days upregulated distal tubular NCC resulting in increased Na^+^ reabsorption and increased blood Na^+^ concentrations (152). Using Hyp mice with elevated FGF23, studies have also shown that these mice have increased distal tubular Na^+^ uptake and membrane abundance of NCC (152). It was explored whether the effects of FGF23 on NCC expression in kidney may potentially drive physiological changes including hypertension and heart hypertrophy in a αKlotho-dependent manner. The inhibition of NCC using chlorothiazide abrogated FGF23-induced heart hypertrophy suggesting that FGF23 may act as a potential regulator of renal Na^+^ reabsorption with downstream consequences, although patients with FGF23-related gain or loss of function mutations primarily show more severe defects in phosphate metabolism. Another mineral that may be regulated by FGF23 in distal tubule is calcium (153). In this regard, studies have shown that renal calcium reabsorption and renal membrane abundance of TRPV5 are reduced in *Fgf23*^−/−^/VDR^Δ/Δ^ and *Kl*^−/−^/VDR^Δ/Δ^ double mutant mice (153).

### Renal FGF23 Signal Transduction

Although FGFR1c, 3c, and 4 are ubiquitously expressed, Klotho expression is predominantly expressed in specific tissues such as kidney renal tubules, parathyroid gland, and choroid plexus of brain, suggesting that these organs are the physiological targets for FGF23-mediated endocrine actions (39, 154, 155). FGF23 preferentially binds to FGFR1c and Klotho, and this complex initiates FGFR1c signal transduction via the cytoplasmic adaptor FRS2α (156), which activates the FRS2α/Ras/MAPK pathway (157) (Figure 3). *In vitro* studies using human embryonic kidney cells (HEK293), which endogenously express FGFRs but not Klotho (158), showed that the presence of soluble Klotho (sKL) or membrane-bound Klotho (mKL) is required for FGF23-induced MAPK activity, which was assessed by pERK1/2 induction and EGR1 mRNA expression (157, 159). Although initial studies suggested that mKL and sKL share a common function of mediating FGF23-induced FRS2α/Ras/MAPK signaling, recent findings suggested that FGF23 responses were quantitatively different depending on mKL or sKL availability (159). *In vivo* studies, potentially using genetic targeting to isolate the biological effects of mKL from sKL will be required to deepen our understanding of these interactions.

In the absence of Klotho, in HEK293 cells, FGF23 alone can activate pPLCγ and pAKT, and these activities are completely neutralized by the presence of Klotho (159, 160). These studies further suggested that FGF23 preferentially induced FGFR1c signaling *via* Klotho. However, in the absence of Klotho, high concentrations of FGF23 can activate FGFR4 (157). The activation of FGFR4 was shown to induce PLCγ-catalyzed production of diacylglycerol and inositol 1,4,5-triphosphate that increased cytoplasmic calcium levels, thereby activating several calcium-sensing signal mediators, including the protein phosphatase calcineurin (157). The activation of calcineurin dephosphorylates the transcription factor NFAT, which permits its translocation into the nucleus to modulate the expression of specific target genes (161). This FGFR4-mediated effect may play a key role in cardiac hypertrophy through FGFR4 during highly elevated FGF23 in CKD (157, 162).

The phosphaturic action of FGF23 in kidney proximal tubule and actions in the distal tubule may occur primarily through FGFR1c, the main “phosphaturic” FGFR expressed in both segments and colocalized with Klotho (123, 129). A *C*-terminal FGF23 peptide antagonist has been developed recently to block FGF23 signaling by inhibiting tyrosine phosphorylation of FRS2α and downstream activation of the MAPK cascades (34). Studies in Hyp mice using this novel peptide confirmed that the inhibition of FGF23 signaling in kidney upregulates the expression of the sodium-phosphate cotransporters Npt2a and Npt2c, coupled with the alleviation of the observed hypophosphatemia (34). In a mouse model of CKD, this FGF23 antagonist peptide has been shown to rescue the prevailing anemia (163).

Beside the effects of FGF23 on phosphate homeostasis, FGF23 signaling has been described to promote renal calcium reabsorption through the TRPV5 channel. Indeed, the apical membrane abundance of TRPV5 in renal distal tubules could be regulated by the binding of FGF23 to FGFR-Klotho complexes which activated a signaling pathway implicating ERK1/2, SGK1, and WNK4. This signaling pathway led to the increase of intracellular transport of fully glycosylated TRPV5 from the Golgi apparatus to the apical plasma membrane, thus decreasing the renal loss of calcium (153). In distal convoluted tubule, the ERK1/2-SGK1-WNK4 signaling pathway leads to WNK4 serine phosphorylation at residue 71 and kinase activation. FGF23 promoted the physical interaction between NCC and WNK4, increasing NCC membrane abundance, and would promote sodium reabsorption (152). Additional studies of the actions of FGF23 and Klotho specifically within the kidney distal tubule are required to determine the full extent of kidney FGF23 bioactivity.

## Conclusion

The hormone FGF23 is mainly produced by osteocytes with the ability to target distant organs such as kidney. In late osteoblasts and osteocytes, FGF23 can be upregulated by elevated phosphate, anemia, inflammation, PTH and 1,25D; and downregulated by hypophosphatemia, insulin, and insulin-like growth factor 1. Although not covered here, studies have shown that FGF23 can be produced at lower levels by other cells such as immune cells, bone marrow erythroid cells and other tissues such as liver in response to diverse stimuli (106, 164). The posttranslational modifications of FGF23 protein *via O*-glycosylation and phosphorylation controls the proteolytic cleavage of mature FGF23 protein which dictates biologically active FGF23 concentrations.

The binding of FGF23 to FGFR1-Klotho complexes in the kidney has been shown to induce a signaling cascade through MAPK which controls mineral metabolism. The signals induced by FGF23 in kidney downregulate the expression of Npt2a/c leading to decreased phosphate reabsorption in proximal tubules, and upregulation of TPRV5 and NCC, potentially promoting calcium and sodium reabsorption, respectively, in the distal tubule. Alterations of FGF23 expression in osteocytes, FGF23 processing, and FGF23 activity cause severe endocrine pathologies resulting in rare and common diseases. Thus, further understanding the mechanisms controlling FGF23 production in osteocytes and bioactivity in kidney will lead to improved patient outcomes.

In summary, although much is known regarding FGF23 regulation and actions, gaps in our knowledge exist. These include the potential contributions of bone cells such as osteoblasts and osteoclasts in the regulation of osteocytic FGF23, and it remains unclear whether aged osteocytes (mature cells and deeply embedded in the mineralized bone matrix) are more effective in terms of upregulating FGF23 in response to physiological and pathological changes vs. early osteocytes. Finally, whether FGF23 can target other cell-types in the kidney beyond its defined actions on proximal and distal tubules remains unknown, thus future investigation could examine the effects of FGF23 on renal immune cells such as macrophages and regulatory T cells, critical for the control of renal inflammation and kidney remodeling during acute kidney injury and CKD.

## Author Contributions

RA, PN, MN, and KW wrote and edited the manuscript and figures. All authors contributed to the article and approved the submitted version.

## Conflict of Interest

KW receives royalties from Kyowa-Hakko-Kirin Pharmaceutics, Inc., and research funding from Akebia-Keryx, both of which had no part in the study design, data collection and analysis, decision to publish, or preparation of this manuscript. The remaining authors declare that the research was conducted in the absence of any commercial or financial relationships that could be construed as a potential conflict of interest.
